# Gender disparities in the socio-economic burden of HIV/AIDS among patients receiving care in an HIV clinic in Lagos, Nigeria

**DOI:** 10.4314/ahs.v22i4.54

**Published:** 2022-12

**Authors:** Titilope O Charles-Eromosele, Oluchi J Kanma-Okafor, Adekemi O Sekoni, Bolatito O Olopade, Oluwarotimi B Olopade, Ekanem E Ekanem

**Affiliations:** 1 Department of Community Health and Primary Care, Lagos University Teaching Hospital, Lagos Nigeria; 2 Department of Community Health and Primary Care, College of Medicine, University of Lagos, Lagos Nigeria; 3 Department of Medical Microbiology and Parasitology, Obafemi Awolowo University, Ile-Ife; 4 Department of Medicine, Lagos University Teaching Hospital, Lagos Nigeria

**Keywords:** HIV/AIDS, gender disparities, women, Lagos, Nigeria

## Abstract

**Background:**

In sub-Saharan Africa, women are bearing a heavier burden than men in terms of rate of infection and socio-economic impact of HIV/AIDS. This study was aimed at assessing gender disparities in the socio-economic burden of HIV/AIDS.

**Methods:**

This descriptive cross-sectional study was conducted among 422 HIV-positive adult patients attending an HIV clinic in Lagos, Nigeria, selected by multi-stage sampling and interviewed using a pretested, semi-structured questionnaire. Bivariate analysis was used to assess how the socioeconomic constructs differed by gender.

**Results:**

This study revealed that females suffered more of the socio-economic consequences of having HIV/AIDS than males; cruelty and isolation were significantly higher among the females (p<0.0001), more females (50.0%) were discriminated against at the workplace compared to males (32.1%) (p=0.005), physical abuse (p=0.002) and extortion (p=0.029) were experienced by more of the females than the males. Also, the cost of care outside of antiretroviral therapy was significantly higher among the females (p= 0.002).

**Conclusion:**

Quantifying the social and economic disparities between HIV-infected men and women has shown that the burden is by far higher among women than men. Focused interventions are therefore needed to control the spread of the disease and improve the quality of life of HIV-infected women.

## Introduction

The Human immunodeficiency virus (HIV) infection is a major cause of morbidity and mortality worldwide.^1^ This disease places a high socio-economic burden on countries and individuals by dwindling the development gains of nations, thereby increasing poverty, especially in the poor countries of the world. Globally, 38.0 million [31.6 million–44.5 million] people were living with HIV in 2019. Sub-Saharan Africa remains most severely affected, with about 25.6 million people living with HIV in 2019, accounting for over two-thirds of the total global prevalence.^3^

The impact of HIV/AIDS has been felt not only in terms of increased mortality and morbidity but also in the socio-economic sphere since the disease disproportionately affects young adults and those in the productive age groups.^2^ Strong empirical evidence suggests that the disease has had an adverse impact on households and firms as well as the macroeconomy of affected countries.^2^ In many sub-Saharan African countries, women and girls are bearing a heavier burden than men in terms of a higher rate of HIV infection, the stigmatization that results from HIV/AIDS and the socio-economic burden of family support and care.^4^,^5^,^6^ Nigeria accounts for the second largest HIV epidemic in the world and has one of the highest rates of new infection in sub-Saharan Africa. Nigeria bears about 5% of the global burden of HIV. As of 2020, about 1.7 million people were living with HIV in Nigeria with only about 86% of people living with HIV getting access to treatment. 7 There is poor access to treatment and care for people living with HIV/AIDS in Nigeria thereby creating a huge socio-economic burden. In the general population, the infection very often results in unemployment, loss of income, rejection (by spouse or partner, family or community), disruption in interpersonal relationships due to guilt and shame, taboo, and social stigmatization.^7^ Society naturally makes women, by their gender roles, carry the burden of care when their close relatives are sick.^8^ Most often, these women are neglected and isolated as there is usually nobody to care for them when they too are sick. They are also more vulnerable economically as they are less financially empowered compared to men.^9^ Cultural practices such as inheritance laws which restrict the transfer of wealth and property to males only worsen the situation.^9^ Females are less educated compared to males, especially in the developing countries. 9 They, therefore, have limited access to HIV/AIDS information and services, and are less informed about the management of the disease.^9^ It is important to note that women, being more affected by HIV/AIDS than men, increase the number of children affected via mother-to-child transmission.

It is important to assess the impact of HIV in driving poverty. Moreover, though studies have described the gender disparity in HIV prevalence, the specific social burden and the poverty imposed on females living with HIV is less explored. The ‘feminization of HIV/AIDS draws attention to studying how males and females are differentially affected by HIV infection. This study was therefore conducted to assess gender disparities in the socio-economic burden of HIV/AIDS among patients who receive care and other supportive services at a designated HIV/AIDS clinic in Lagos, Nigeria.

## Methods

This was a descriptive cross-sectional study conducted among HIV/AIDS patients, 15 years and above, attending the HIV clinic at the Lagos University Teaching Hospital in Lagos, a metropolitan city in Western Nigeria, for at least one year. A sample size of 422 was calculated using the Cochrane formula. The study participants were selected by multistage sampling. In the first stage, by simple random sampling using the balloting method, ten clinic days were selected out of 20 clinic days in the period of four weeks during data collection. In the next stage, a total of 42 respondents were selected on clinic days one to eight while 43 respondents were selected on clinic days nine and ten, by systematic random sampling. The selection of participants was based on the patients' arrival at the clinic on each clinic day. An average of 90 patients attends the clinic on each clinic day. The first patient to arrive at the clinic was enrolled in the study. Every other patient arriving at the clinic after the first patient was enrolled in the study. Enrollment of participants continued till the required number (42 or 43) was attained. Data were collected using a pretested, interviewer-administered questionnaire consisting of 47 items in six sections designed to collect socio-demographic information, information on the respondents' HIV/AIDS status, HIV-related stigmatization and discrimination, respondents' experience of physical violence, caregiver perspectives of the burden of HIV/AIDS, the economic effect of respondents' HIV status such as the effect on income-earning capability and the cost of care.

Ethical approval was obtained from the Health Research Ethics Committee (HREC) of the Lagos University Teaching Hospital (HREC approval number: ADM/DCST/HREC1395). Written informed consent was obtained from each respondent before an interview. The purpose of the study was clearly explained to the respondents and confidentiality was ensured by anonymity. secure locking in cabinets and pass-wording of the data file. Participants were free to withdraw from the study at any time without consequences.

Data were analyzed using SPSS version 26. Bivariate analyses using the chi-square and independent t-tests were performed in determining the variables that were significantly different between the male and the female patients. A p-value ≤0.05 was considered statistically significant.

## Results

The gender distribution of respondents revealed male to female ratio of 2:3. The mean age of respondents was 38.88±8.97 years. The greater proportion of the respondents (39.6%, male; 40.3%. female) were between 35–44 years of age. The age distribution between the men and women was comparable. The majority of the respondents (81.8%) were Christians. More females than males had higher levels of education; 31.2% of the females compared to 11.8% of the males had tertiary education, while 10.3% of the females compared with 6.0% of the males had a postgraduate degree. A small proportion (6.3%) of the females compared with 20.7% of the males had only primary education. This difference in the level of education between both groups was statistically significant (p<0.001). Though the majority (85.8%) of the respondents were employed (92.3% of the males and 81.4% of the females) and 14.2% were unemployed (7.7% of the males and 18.6% of the females) and the difference was statistically significant (p=0.002) About three quarters (73.5%) of the respondents were married, a greater proportion of the males (89.4%) than the females (62.8%) were married, a statistically significant difference (p<0.001) (Table 1).

**Table 1:** Socio-demographic characteristics of the respondents

Variable	Male n=169 Freq (%)	Female n=253 Freq (%)	Total N=422 Freq (%)	X^2^	P-value
**Age (years)**					
15–24yrs	3(1.8)	5(2.0)	8 (1.9)		0.512*
25–34yrs	49(29.0)	89(35.2)	138(32.7)		
35–44yrs	67(39.6)	102(40.3)	169(40.0)		
45–54yrs	40(23.7)	46(18.2)	86(20.4)		
55–64yrs	10(5.9)	11(4.3)	21(5.0)		
**Mean age ±SD**	39.80±9.16	38.27±8.80	38.88±8.97	T=1.02	0.307
**Religion**					
Christianity	131(77.5)	214(84.6)	345(81.8)		0.171*
Islam	35(20.7)	36(14.2)	71(16.8)		
Others	3(1.8)	3(1.2)	6 (1.4)		
**Highest level of** **education attained**					
Primary	35(20.7)	16(6.3 )	51(12.1 )	37.44	<0.001
Secondary	104(61.5)	132(52.2)	236(55.9)		
Tertiary	20(11.8)	79(31.2)	99(23.5)		
Post-graduate	10(6.0)	26(10.3)	36(8.5)		
**Employment status**					
Employed	156(92.3)	206(81.4)	362(85.8)	9.84	0.002
Unemployed	13(7.7)	47(18.6)	60(14.2)		
**Marital status**					
Single	11(6.5)	52(20.6 )	63(14.9)	36.62	<0.001
Married	151(89.4)	159(62.8)	310(73.5)		
Separated/divorced/ Widowed	7(4.1)	42(16.6)	49(11.6)		

* Fisher's exact p-value

Even though more females (172, 68.8%) than males (108, 63.9%) had disclosed their HIV status immediately their status was known to them, the two groups were not significantly different (p= 0.385). None of the males experienced cruelty and social isolation, while a third (35.6%) of the females did. The difference was statistically significant (p<0.001). Less than a third (28.2 %) of all the respondents experienced social discrimination by family members (males 30.2%, females 26.9%, p<0.001), 63% were asked to leave home (males 21.3%, females 10.9%), more females (8.3%) than males (1.8%) were deprived of their basic necessities, 6.4% were neglected (males 6.5%, females 6.3%), while 1.2% of the respondents, all females, experienced abuse by family members. Discrimination at the workplace was significantly higher among the females (p=0.001). Physical abuse or beating was less prevalent (4.3%) though significantly different between both groups (p=0.002). None of the males reported ever having their belongings forcefully taken from them compared to 1.7% of the females (p=0.045) (Table 2).

**Table 2:** Status disclosure and social discrimination by gender

Variable	Gender		Total Freq (%) N=422	X^2^	P-value
	Male Freq (%) n=169	Female Freq (%) n=253			
**Early/immediate disclosure of HIV** **status**					
**Yes**	108(63.9)	172(68)	280(66.4)	0.76	0.385
**No**	61(36.1)	81(32)	142(33.6)		
**Cruelty and isolation**					
**Yes**	0(0)	80(31.6)	80(19)		<0.001*
**No**	169(100)	173(35.6)	342(79.4)		
**Experience of discrimination**					
**-By family members**					
**Yes**	**51(30.2)**	**68(26.9)**	**119(28.2)**		
- Asked to leave home	37(21.3)	26(10.9)	63(14.9)		<0.001*
- Neglected	11(6.5)	16(6.3)	27(6.4)		
- Deprived	3(1.8)	21(8.3)	24(5.7)		
- Physically Abused	0(0)	5(2)	5(1.2)		
**No**	**118(69.8)**	**185(73.1)**	**303(71.8)**		
**-At the workplace** **(male, n=156; female, n=206; total,** **n=362)**					
**Yes**	50(32.1)	103(50.0)	153(42.3)	11.72	0.001
**No**	106(67.9)	103(50.0)	209(57.7)		
**Physical abuse or beating**					
**Yes**	1(0.6)	17(6.7)	18(4.3)		0.002*
**No**	168(99.4)	236(93.3)	404(95.7)		
**Belongings forcefully taken**					
**Yes**	0(0)	7(2.8)	7(1.7)		0.045*
**No**	169(100)	246(97.2)	415(98.3)		

* Fishers exact p-value

A greater proportion of the respondents had an average monthly income of less than 75 000 Naira, however, the average monthly income was comparable between both groups (p=0.001). A total of 123(29.1%) respondents lost some income due to their HIV status. While 19(11.2%) of the male respondents and 104(41.1%) of the females lost income monthly due to HIV/AIDS, the majority of those who lost income lost less than 5000 Naira (about 12.12 United States Dollar). All the male respondents who lost income, lost less than 5000 Naira while about half of the females lost over 5,000 Naira monthly. Loss of income monthly due to HIV/AIDS was significantly higher among the female respondents (p<0.001). About half (48.9%) of the respondents who were in formal employment received support from their employers; a higher proportion of the females (51.4%) than the males (37.5%) received any form of support from their employers as a result of their HIV status. This difference was however not statistically significant (p=0.218). A small proportion (14.2%) of the respondents received additional financial support, mostly from family members (70.0%), the females however received significantly more financial support than the males (p=0.016). The monthly cost of care was significantly higher for more of the females than the males (p<0.001). About two-thirds (65.1%) of the male respondents spent less than 5,000 Naira on the treatment of HIV/AIDS monthly compared to 54.5% of the females. Also, while none of the males spent beyond 10000 Naira on their treatment about a tenth (9.1%) of the females incurred a cost of 10000-100000 Naira. To be able to cope with the financial burden of HIV/AIDS, significantly more females than males (p=0.048) had to take additional measures like using personal savings (males, 35.5%; females, 40.3%), borrowing from family/friends (males, 27.8%; females, 27.7%), selling off personal assets (males, 0%; females, 2.8%), borrowing from their employer (males,11.8%; females, 4.7%), seeking support from NGOs (males,11.8%; females, 12.6%), discontinuing HIV/AIDS treatment (males, 0.6%; females, 1.2%) and other measures such as taking on additional jobs, relying on donations from faith-based organizations and loans from social support groups (males,12.4%; females, 10.7%) (Table 3).

**Table 3:** Respondents' economic situation by gender

Variable	Gender		Total Freq (%) N=422	X^2^	P-value
	Male Freq (%) n=169	Female Freq (%) n=253			
**Average monthly income (in Naira)**					
5,000–40,000	66(39.1)	100(39.5)	166(39.3)	0.45	0.930
>40,000-less than	75,000	60(35.5)	94(37.2)	154(36.5)	
75,000–100,000	32(18.9)	46(18.2)	78(18.5)		
>100,000	11(6.5)	13(5.1)	24(5.7)		
**Loses income monthly (in Naira)**	**19(11.2)**	**104(41.1)**	**123(29.1)**		
Less than	5,000	19(100.0)	57(54.8)	76(61.8)	<0.001*
5,000–10,000	0(0.0)	28(26.9)	28(22.8)		
>10,000	0(0,0)	19(18.3)	19(15.4)		
**Employer support (if in formal employment)** **(male, n=24, female, n=107, total, n=131)**					
Yes	9(37.5)	55(51.4)	64(48.9)	1.52	0.218
No	15(62.5)	52(48.6)	67(51.1)		
**Additional sources of financial support**	**23(13.6)**	**37(14.6)**	**60(14.2)**		
Family support	19(82.6)	23(62.2)	42(70.0)		0.016*
NGO	3(13)	2(5.4)	5(8.3)		
Community	1(4.3)	2(5.4)	3(5.0)		
Others*	0(0)	10(27)	10(16.7)		
**Monthly cost of care (in Naira)**					
Less than 5,000	110(65.1)	138(54.5)	248(58.8)		<0.001*
5,000–10,000	59(34.9)	92(36.4)	151(35.8)		
10,000–50,000	0(0)	16(6.3)	16(3.8)		
50,000–100,000	0(0)	6(2.4)	6(1.4)		
>100,000	0(0)	1(0.4)	1(0.2)		
**Methods of coping with the increasing** **financial burden**					
Using personal savings	60(35.5)	102(40.3)	162(38.4)		0.048*
Borrowing from family/friends	47(27.8)	70(27.7)	117(27.7)		
Sale of personal assets	0(0)	7(2.8)	7(1.7)		
Loan from employer	20(11.8)	12(4.7)	32(7.6)		
Seeking NGO support	20(11.8)	32(12.6)	52(12.3)		
Discontinuing treatment	1(0.6)	3(1.2)	4(0.9)		
Others#	21(12.4)	27(10.7)	48(11.4)		

* Fishers exact p-value

#Churches, mosques and private organizations, #Taking up additional jobs, donations from churches, donations from mosques, loans from social support groups, etc.

**Figure 1:**
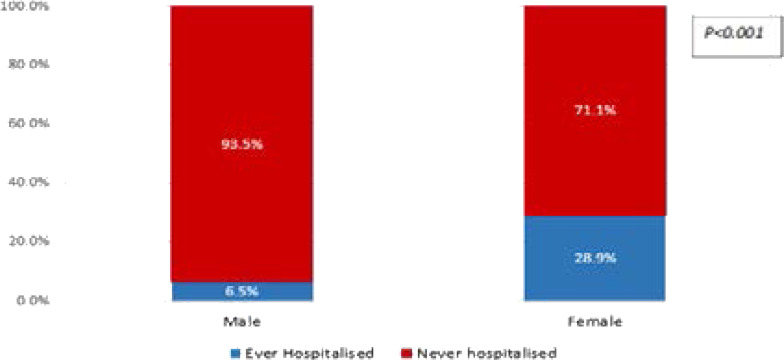
Gender differences in hospitalization experience

Less than one-tenth (6.5%) of the male respondents compared to about a quarter (28.9%) of the female respondents had been hospitalized since infection with HIV/AIDS. Hospitalization was significantly higher in females (p=0.000) as shown in figure 1.

**Figure 2:**
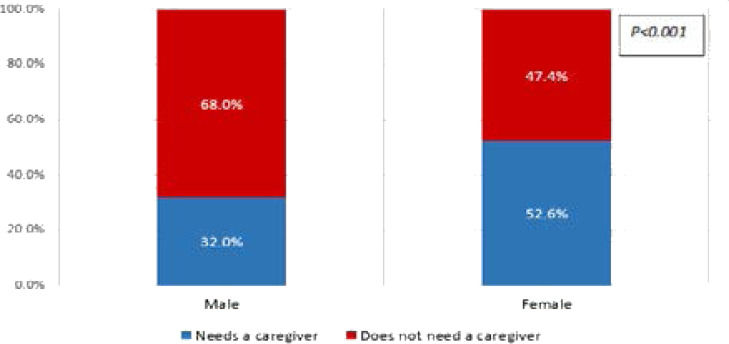
Gender differences in the need for a caregiver

Significantly more females (52.6%) compared to the males (32.0%) needed additional care requiring a caregiver as a result of ill health (p<0.001) (Figure 2).

## Discussion

This study examined the differences in the socio-economic burden of HIV/AIDS between men and women and had one main finding which is that compared with men, women were significantly more disadvantaged socially and financially and required more support despite their higher levels of education. These findings were highly statistically significant, indicating the worse status of women.

Though in our study age was comparable between both genders, across Africa women of a younger age group (20–39 years) were more likely to be HIV-infected than men of the same age group, particularly in Congo Brazzaville, Zambia, and Zimbabwe. At variance with our study, it has been found that women are more likely to be uneducated, unemployed, and hence poorer than men, which in the first place places them in the position of being at the risk of getting infected with HIV because they are predisposed to transactional sexual exchanges. Sexually active women may have an increased vulnerability to HIV because of the economic inequality between males and females. Our study confirmed this inequality by the significantly higher proportion of the unemployed among the women (p=0.002), even though we found that the women were better educated (p<0.001). On the contrary, a study using the Demographic and Health Survey of several Sub-Saharan African (SSA) countries found that compared to men, fewer women had secondary or higher education (11.1 % versus 31.9 % in Guinea; 25.3 % versus 36.6 % in Uganda), but supported the finding that women were more frequently unemployed compared to men.^17^

Our study found a significant difference in marital status as more of the women were single or divorced. A larger multinational survey among adults with HIV found that across SSA, females were more likely to be married (e.g., 76.7 % versus 63.8 % in Sierra Leone; 62.8 % versus 50 % in Cameroon) and to be separated/divorced/widowed (e.g., 9.1 % versus 5.4 % in Ghana; 18.4 % versus 5.5 % in Mozambique) compared to males.^17^ A study in Kenya suggested that marital status may be an important factor related to gender inequalities in HIV/AIDS prevalence showing a two-fold risk of HIV infection among unmarried women compared to unmarried men.

While stigma and discrimination were a general finding in some HIV patients in Lagos,^17^ in our study social discrimination was experienced by more women compared to men. In India, women may often be treated badly, be accused of infidelity and be blamed for their husband's illness. 6 However, contrary to our study more women were discriminated against by their own families. 6 This might be due to the lower status of women in India, whereas women of Yoruba descent in Nigeria, among whom this study was conducted, bear a higher status socially.

Despite recent public enlightenment campaigns in many countries, discrimination at home or in the workplace has persisted. In Delhi and Manipur, India 10.4% of HIV-infected individuals experienced discrimination at work, 6 much lower than was found in our study (42.3%). More women than men with HIV/AIDS experience physical abuse or beating. In Kenya, 19% of women reported intimate-partner violence because of their HIV status, and in the USA, a WHO study found that 20.5% of women living with HIV reported experiencing physical abuse because of their HIV status. In India, HIV-positive women whose husbands die are often evicted from their homes, with one study putting the proportion of women experiencing this as high as 91%. Similarly, in Vietnam, HIV-positive women have been beaten, ejected from their homes, or had their children removed from them by their relatives.

The current study showed that monthly income loss due to HIV/AIDS was significantly higher in females (p=0.001). In some agricultural and farming communities in Nigeria, 85% of the farm women with HIV/AIDS experienced a reduction in their family income while 56% of the women lost one or more of their family's assets as a result of the HIV/AIDS epidemic in their communities. Overall, loss of income was reported by 60% of women with HIV/AIDS in Eastern Nigeria.^21^ The loss of income worsens the HIV/AIDS burden to the individual, meaning that they do not have the resources to provide all they need to sustain their health further worsening their disease outcome.

The government subsidy on antiretroviral drugs in many countries including Nigeria, through the National Antiretroviral program, has kept the cost of care at a minimum. 25 Still the illness burden of HIV/AIDS is substantially heavy on HIV households as compared to non-HIV households in Nigeria and India. A bi-regional study in Nigeria among households affected by HIV reported direct private healthcare costs and indirect income loss of over half (56%) of annual income per capita, with only 10% of the cost of HIV care being accounted for by government subsidies. This could potentially interrupt the targeted HIV care continuum of HIV-infected individuals.

Even though our study found that women carry a greater burden regarding the economic and social burden of HIV care, men equally have some challenges regarding HIV treatment and care; outcomes of care among males are directly impacted by men's under-representation in HIV testing, treatment, and care. , , , , Interventions have focused mainly on women however scale-up efforts are hindered by the poor health-seeking behaviors of men, such that men tend to access ART at a later disease stage than women, and have a higher risk of mortality according to some cohort studies conducted in sub-Saharan Africa. , Many factors make the cost of care and the number of hospitalizations higher among the females; some of the clinical manifestations of HIV infection that are specific to women like gynaecological cancers, pelvic infections , and pregnancy , , place HIV-infected women at a biological disadvantage compared to HIV infected men. Interestingly, the efficacy, side effects, safety and metabolism of highly active antiretroviral therapy (HAART) display gender differences such that females are more vulnerable to lactic acidosis, lipodystrophy and disturbances in glucose metabolism compared to males. , , , Socially some very poor women, underprivileged in terms of employment and education, have their freedom limited by the men to whom they are subservient and endure violence.^15^ These correctable and preventable non-biological factors are prevalent and underscore the link between poverty, women and HIV.^15^

In this study, although we identified several factors that were significantly different between the male and female gender, we did not extend our study objectives to identify factors that could explain the gender disparities that we observed. Despite this limitation, we believe our study substantially adds to the literature that addresses gender disparities between women and men and has created room for additional research to identify the independent explanatory factors for gender disparities and to find ways to mediate the identified areas to improve the situation of women living with HIV/AIDS. Our observations in this study have informed our future work in HIV and gendre.

## Conclusion

This study showed that the female patients living with HIV/AIDS faced a greater socio-economic burden than the men. Although we identified gender disparities in prevalence and several socioeconomic contexts, our study did not identify factors that explained them. Qualitative research would likely be beneficial to better understand how to mediate the socioeconomic challenges of HIV-infected women. Enabling socioeconomic strategies that specifically target women living with HIV should be urgently developed.
